# Effect of Manufactured Sand with Different Quality on Chloride Penetration Resistance of High–Strength Recycled Concrete

**DOI:** 10.3390/ma14227101

**Published:** 2021-11-22

**Authors:** Jincai Feng, Chaoqun Dong, Chunhong Chen, Xinjie Wang, Zhongqiu Qian

**Affiliations:** 1 Department of Civil Engineering, Changzhou University, Changzhou 213164, China; cczudcq@163.com (C.D.); chench@cczu.edu.cn (C.C.); wangxinjie@cczu.edu.cn (X.W.); 2 Jiangsu Nigao Science & Technology Co., Ltd., Changzhou 213141, China; qianzhongqiu@126.com

**Keywords:** manufactured sand, chloride penetration resistance, recycled aggregate concrete, high–strength concrete

## Abstract

High–strength manufactured sand recycled aggregate concrete (MSRAC) prepared with manufactured sand (MS) and recycled coarse aggregate (RCA) is an effective way to reduce the consumption of natural aggregate resources and environmental impact of concrete industry. In this study, high–, medium– and low–quality MS, which were commercial MS local to Changzhou and 100% by volume of recycled coarse aggregate, were used to prepare MSRAC. The quality of MS was determined based on stone powder content, methylene blue value (MBV), crushing value and soundness as quality characteristic parameters. The variation laws of compressive strength and chloride penetration resistance of high–strength MSRAC with different rates of replacement and different qualities of MS were explored. The results showed that for medium– and low–quality MS, the compressive strength of the MSRAC increased first and then decreased with increasing rate of replacement. Conversely, for high–quality MS, the compressive strength gradually increased with increasing rate of replacement. The chloride diffusion coefficient of MSRAC increased with decreasing MS quality and increasing rate of replacement. The chloride diffusion coefficient of MSRAC basically met the specifications for 50–year and 100–year design working life when the chloride environmental action was D and E. To prepare high–strength MSRAC, high–quality MS can 100% replace RS (river sand), while rates of replacement of 50–75% for medium–quality MS or 25–50% for low–quality MS are proposed. Scanning Electron Microscope (SEM) images indicated that an appropriate amount of stone powder is able to improve the compressive strength of RAC, but excessive stone powder content and MBV are unfavorable to the compressive strength and chloride penetration resistance of RAC.

## 1. Introduction

The traditional cement concrete industry is unprepossessing due to its high consumption of natural resources and energy, and major environmental damage [1]. In 2020, China produced 2.5 billion m^3^ of concrete, which consumed nearly 1.65 billion tons of sand and gravel, including a large number of natural river sand (RS). As a non–renewable resource, RS is overconsumed. Long–term mining of RS has caused serious threats to the safety and stability of bridges and river banks, the ecological system of origin, and the health of local residents [2,3,4]. In addition, China has promulgated a policy of restriction and prohibition of mining RS. This further leads to less and less high–quality RS being available for the concrete industry, and the rising cost of RS. Therefore, it is imperative to find alternative materials for RS in order to meet the goal of sustainable development in the concrete industry.

Manufactured sand (MS) is a kind of fine aggregate with particle size less than 4.75 mm that is obtained by crushing and screening rocks. It is regarded as a potential alternative material for RS [5]. However, due to its preparation process, MS has defects such as irregular particle shape, unreasonable gradation, high stone powder content, large methylene blue value (MBV), etc. These defects also lead to the performance differences (workability, mechanical properties, and durability) between MS concrete and RS concrete [6,7,8,9]. Many scholars have studied the influence of MS characteristics on the performance of concrete. Shen et al. [10,11] studied the effects of MS of different crushing values, stone powder content and the MBV on performance of C50 MS concrete. It was found that the stone powder content and MBV had the greatest impact on the workability of the concrete. When the stone powder content and MBV remained unchanged, the compressive strength was negatively correlated with the crushing value. Liu et al. [12] studied the influence of MMBV on the durability of medium– and low–strength MS concrete. MMBV is the multiplication of stone powder content and MBV. MMBV has shown an effect on the workability of medium– and low–strength concrete. With increasing MMBV, concrete workability decreases. The compressive strength and chloride penetration resistance of medium–strength concrete were more affected than those of low–strength concrete. Chen et al. [13] studied the coupling effect of different stone powder content and MBV in the performance of C40 concrete, and they calculated the coupling coefficient of stone powder content and MBV on concrete performance. Li et al. [14] studied the effect of stone powder content on the durability of MS fully replaced medium–strength marine concrete. MS with low crushing value and MBV was selected to prepare marine concrete. The optimum content of stone powder was about 7.5%. Li et al. [15] used MS of different crushing value and stone powder content to prepare pavement concrete. The results of research showed that with increasing stone powder content, the compressive strength of pavement concrete improved. The lower the crushing value, the higher the compressive strength. To sum up, the MS characteristics have significant effects on the mechanical properties and durability of low– and medium–strength concrete. However, there are few studies on the effects of MS characteristics on performance of high–strength concrete.

In recent years, the market share of MS has been increasing [16]. Hence, it is generally believed that MS is a low–quality substitute for RS and is only suitable for the preparation of medium– and low–strength concrete [17]. This can also be found from the above studies. However, Shen et al. [18] proved that MS with low crushing value, stone powder content, and MBV can be used to completely replace RS in the preparation of ultra–high–strength concrete, and the compressive strength at 28 d exceeded 130 MPa. This indicates that MS has the potential to be used to prepare high–strength concrete. In fact, on the basis of the main quality characteristic parameters of MS, including stone powder content, MBV, crushing value and soundness, the quality of MS can be divided into categories I, II and III, corresponding to high, medium and low quality, respectively, according to the Chinese standard [19]. Table 1 presents the quality of the MS used in some studies according to the Chinese standard. It can be found that in the past, most studies changed one or two characteristic quality parameters to study their influence on concrete performance. However, they did not reckon with the influence of overall MS quality on concrete performance. The category of a certain quality characteristic parameter cannot be used to determine the overall quality of MS. Hence, the overall quality of MS needs to be comprehensively determined on the basis of multiple quality characteristic parameters. As shown in Table 1, the stone powder content, crushing value, and MBV of the MS used in reference [18] are determined to be class I. The MS can be regarded as being of high quality, and is able to completely replace RS in the preparation of high–strength concrete. On the other hand, for medium– and low–quality MS, the optimum rate of replacement for the preparation of high–strength concrete remains unclear. In addition, few studies about manufactured sand recycled aggregate concrete (MSRAC) have been reported, especially with respect to the effect of different levels of MS quality on the durability of high–strength RAC.

The research and use of RAC in China started later than that in developed countries. At present, the use rate of waste concrete in China is low, at only about 10%. A large amount of waste concrete has piled up, resulting in serious land pollution [20,21,22]. The application of RAC contributes to solving this problem. Many studies have investigated the durability of RAC [23,24,25]. Chloride penetration resistance is one of the most important durability properties of RAC. Mata et al. [26] found that RAC had higher chloride permeability than NAC. The lower resistance of RAC to chloride ion penetration compared with NAC can be attributed to the old interfacial transition zone and cement paste attached to the recycled aggregate, which make RAC more penetrable than NAC. Due to the presence of old mortar, RAC has multiple interfaces, such as new mortar–old mortar, new mortar–aggregate, and old mortar–aggregate, leading to RAC having more complex and heterogeneous material properties. The old mortar layer is the fundamental reason for the poor durability of RAC [27]. Loose and porous old mortar and the weak interfacial transition zone provide a large number of channels for chloride ions to enter the concrete [28,29]. Chloride ions are present and migrate in the concrete in the free and chemically or physically bound states, deteriorating the interface structure of the concrete and causing the corrosion of steel bars. This leads to the cracking and destruction of reinforced concrete structures, and continuously reduces the bearing capacity of concrete structures. Especially in concrete structures exposed to marine environments, deicing salt environments and coastal underground environments, the chloride penetration resistance of the concrete protective layer is particularly important.

Therefore, in order to improve the use rate of MS and promote the application of medium– and low–quality MS in chloride ion environments, it is necessary to clarify the influence of different levels of MS quality on the chloride penetration resistance of high–strength RAC at different rates of replacement. In this experiment, high–, medium– and low–quality MS are used to prepare MSRAC. The effects of MS of different quality on the compressive strength and chloride penetration resistance of high–strength RAC with different rates of replacement are explored in order to define the optimum rate of replacement of MS of different quality. The microstructure of MSRAC was observed using Scanning Electron Microscope (SEM) technology to clarify the influence of different levels of MS quality on MSRAC.

## 2. Experimental Section

### 2.1. Materials

Type P.O. 52.5 Portland cement was used. Commercially available fly ash and silica fume were used as mineral additives. The composition of fly ash and silica is shown in Table 2. The properties of the cement are shown in Table 3.

**Figure 1:**
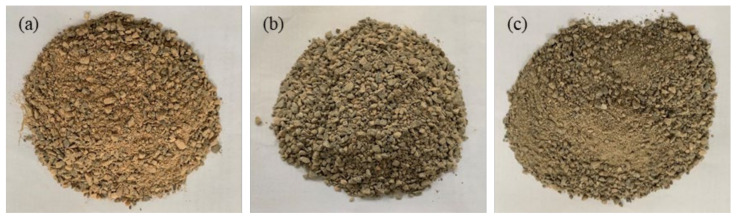
Three levels of MS quality: (**a**) MS1; (**b**) MS2; (**c**) MS3.

**Figure 2:**
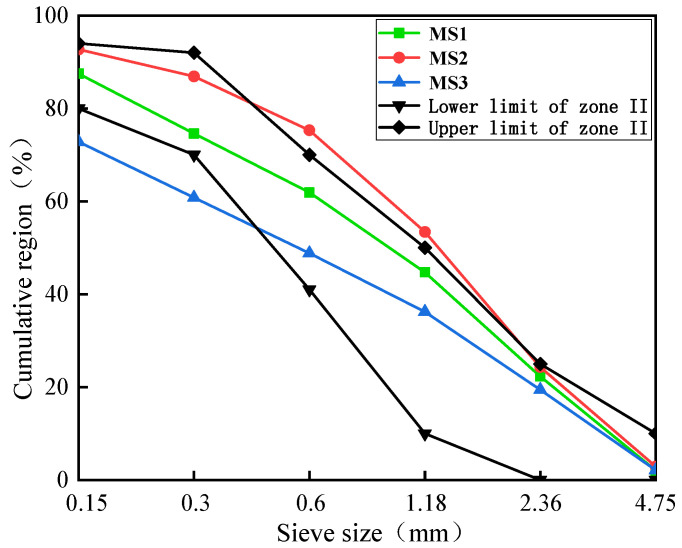
Gradation curves of three MS.

**Figure 3:**
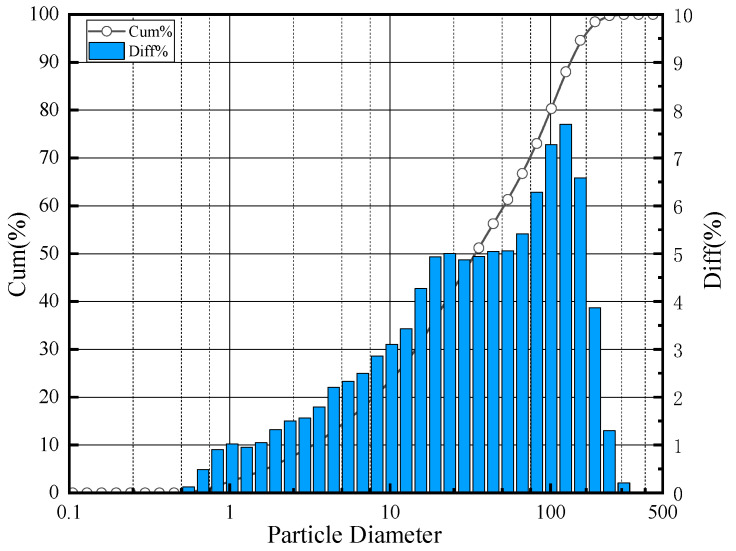
Particle size distribution of stone powder.

**Figure 4:**
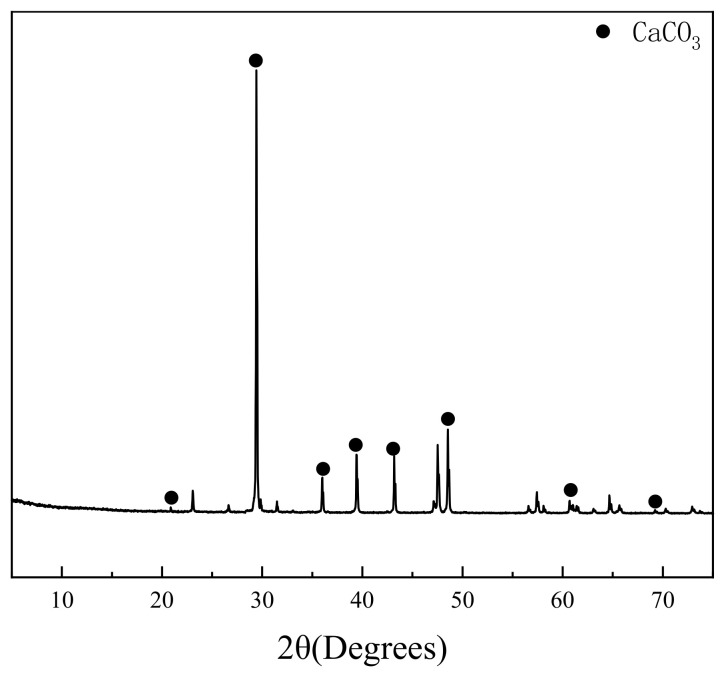
XRD pattern of stone powder.

River sand and three different levels of MS quality were used, which were denoted as RS, MS1, MS2 and MS3, respectively (Figure 1). The MS were commercially manufactured locally in Changzhou. The characteristics of the MS were tested according to GB/T 14684–2011 [19]. The specific results are shown in Table 3. The gradation curve of MS is shown in Figure 2. Stone powder was used to adjust the stone powder content of the MS. The particle size distribution and XRD results of the stone powder are shown in Figure 3 and Figure 4, respectively. Stone powder content, MBV, crushing value and soundness were selected as parameters for comprehensively determining the quality of the MS. It can be seen from Table 4 that, according to GB/T 14684–2011 [19], MS1, MS2 and MS3 can be categorized as high–quality, medium–quality and low–quality MS. Because its MBV > 1.4 and it has a high stone powder content, MS3 can even be classified as a substandard product.

The coarse aggregate consisted of a 5–20 mm continuous gradation of RCA, which was tested according to GB/T 25177–2010 [30]. The specific results are shown in Table 5.

The admixture was a polycarboxylate superplasticizer, and the water reduction rate was 40%. The mixed water was tap water.

### 2.2. Concrete Mix Design

The gradation of the high–quality MS was reasonable. The values of the quality characteristic parameters—stone powder content, crushing value and soundness—were close to those of natural sand, and the MBV was low. Two rates of replacement of 50% and 100% were selected. The performance of medium–quality MS was slightly worse than that of natural sand, and rates of replacement of 50%, 75% and 100% were selected. The optimum rate of replacement for low–quality MS was the research focus, so four rates of replacement of 25%, 50%, 75% and 100% selected. The designed strength of the concrete was 80 MPa, and the water–binder ratio was 0.25. The amount of additional water was calculated on the basis of the water absorption of RCA and MS. The control group was RAC, which was prepared entirely using RS. To make the performance of RAC comparable, the designed strength, water–binder ratio, and the amount of RCA and cementitious material of the MSRAC were consistent with those in the RAC. The amount of superplasticizer in the RAC was adjusted according to the actual mixing situation, and the amount in MSRAC was the same as in RAC. Table 6 shows details of the proportions in the concrete mixture.

### 2.3. Methods

**Figure 5:**
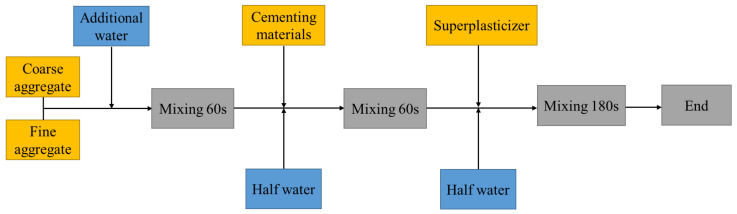
Flow diagram of the concrete secondary mix process.

According to the proportions shown in Table 5, the mixture was mixed with the secondary mixing process proposed by Tam et al. [31]. The specific process used is shown in Figure 5. The fresh concrete was loaded into the mold, vibrated for 10–20 s, and demolded after 24 h. Each sample was placed in a standard curing room at a temperature of 20 ± 2 °C and a relative humidity of 95% for 28 d according to GB/T 50081–2002 [32].

The particle size distribution of the stone powder was determined using a laser granulometer SALD–2201 and without using a solvent. The XRD pattern of the stone powder was determined using a D8 ADVANCE from Bruker, which was used for conventional phase analysis and semi–quantitative analysis of polycrystalline samples, determination and correction of cell parameters, determination of grain size and crystallinity, and indexing of unknown polycrystalline samples. The test angle was 6–75 degrees. The anticathode was a copper target.

The slump of fresh concrete was tested according to GB/T 50080–2016 [33]. Concrete fluidity was observed when performing the concrete slump test. Compressive strength was determined by testing 150 mm × 150 mm × 150 mm cubes according to GB/T50081–2002 [32]. Three test blocks were tested for each proportion.

The chloride penetration resistance was determined by means of the method for testing the rapid chloride ion migration coefficient (RCM) and was represented by the chloride diffusion coefficient according to the GB/ T 50082–2009 [34]. The test block was placed at the bottom of the rubber sleeve, and it was tightened up and down. The rubber sleeve containing the test block was installed in the test tank, and the anode plate was installed. Then, 300 mL 0.3 mol/L NaOH solution was injected into the rubber sleeve, and 12 L 10% NaCl solution was added into the cathode. The continuous voltage of the test was 60 V. The specimen size was ø100 mm × 50 mm. Three test blocks were tested for each proportion.

The contents of free and bound chloride ions were determined according to SL352–2006 [35]. After the RCM test, the specimens were divided into two halves along the radius, and the mortar specimens at depths of 2.5 mm, 7.5 mm, 12.5 mm and 17.5 mm were taken from the bottom of specimens. These mortars were used to test the free and total chloride ions at different concrete depths. The free chloride ion content is represented by $c_{f}$. The total chloride ion content is represented by $c_{t}$. The bound chloride ion content is represented by $c_{b}$ and is equal to $c_{t}$ minus $c_{f}$. The chloride binding capacity is represented by R is equal to $c_{b}$ divided by $c_{f}$.

The SEM observations were performed by means of a Zeiss SU–PRA55 scanning electron microscope. SEM images were obtained under an accelerating voltage of 20 kV. Before the observation, gold was sputtered on the sample.

## 3. Results and Discussion

### 3.1. Workability of High–Strength MSRAC

**Figure 6:**
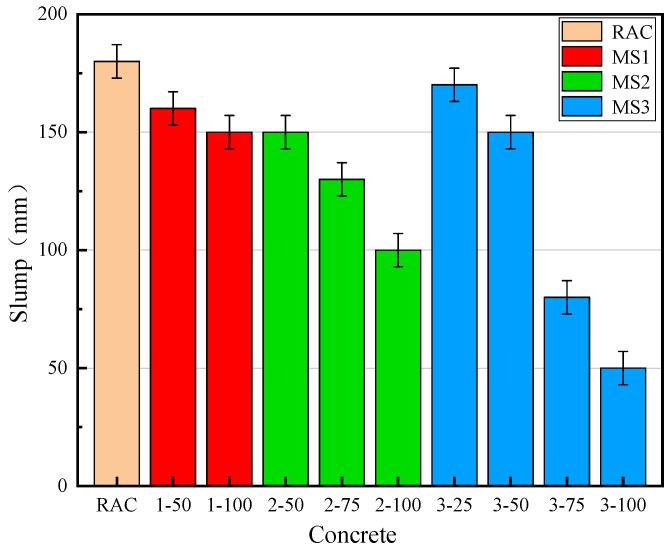
Slump of concrete.

Figure 6 illustrates the results of the slump tests for the fresh concrete. Table 5 shows the fluidity of the fresh concrete. It can be seen that with increasing MS rates of replacement, slump gradually decreased, and the fluidity of the fresh concrete deteriorated. The slump of MSRAC was lower than that of RAC. When the rate of replacement of MS2 exceeded 75%, the slump decreased significantly and the fluidity of the concrete deteriorated. When the rate of replacement of MS3 exceeded 50%, the slump decreased sharply, and the fresh concrete had great viscosity and almost no fluidity. This phenomenon could be due to the quality of the MS. The stone powder content and MBV of the MS were higher than the RS, and increased with decreasing MS quality. Since the specific surface area of stone powder was much higher than that of MS and RS, the water consumption required for wrapping inevitably increased with increasing stone powder content, thereby reducing the workability of the fresh concrete. Studies have shown that MBV is positively correlated with the mud powder content in MS. The higher the mud powder content, the higher the MBV [36]. Mud powder absorbed a large amount of free water, reducing the mixing water. As a result, the slump of the fresh concrete decreased and the workability deteriorated.

### 3.2. 28 d Compressive Strength of High––Strength MSRAC

**Figure 7:**
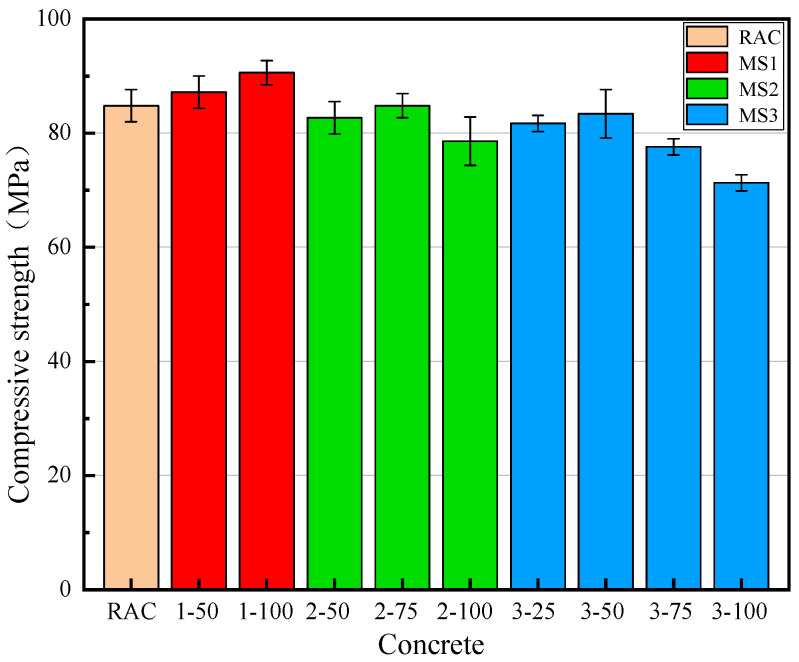
28 d compressive strength of concrete.

Figure 7 shows the 28 d compressive strength of concrete prepared with three different levels of MS quality at different rates of replacement. It can be seen that the compressive strength of MSRAC increased with increasing rate of replacement with MS1. Specifically, the 28 d compressive strengths of concrete containing 50% and 100% MS1 were 2.9% and 6.4% higher than that of RAC, respectively. Due to the low crushing value of MS1, the “interlocking effect” between MS particles took place fully, based on the grade coordination. In addition, an appropriate amount of stone powder in MS1 improved the particle packing density of the concrete, which increased the density of the interface transition zone between the slurry and the RAC and improved the compressive strength [37]. Additionally, the limestone particles in the stone powder played a nucleation role in the formation of Ca(OH)_2_ and C–S–H during the early stage of cement hydration, accelerating the hydration of C3S minerals [38] and improving the compressive strength of the concrete [39].

However, the 28 d compressive strength of the MSRAC first increased, and then subsequently decreased with increasing rates of replacement with MS2 and MS3. The variations in the compressive strength of concrete containing 50% and 75% MS2 were not obvious, but compressive strength decreased sharply when MS2 replacement reached 100%. The compressive strength of the MSRAC with 100% MS2 was 6.2 MPa lower than that of RAC. For the MSRAC with MS3, the variations in compressive strength of the concrete containing 25% and 50% MS3 were not obvious, but compressive strength decreased sharply when MS3 replacement reached 75%. The compressive strengths of concrete containing 75% and 100% MS3 were 11.1% and 16.3% lower than that of RAC, respectively. The quality of MS2 and MS3 was inferior to that of MS1 and RS, leading to a lower compressive strength in the concrete. In addition, the excessive stone powder content in MS2 and MS3 was responsible for higher water absorption and the slump decrease. This led to a lower amount of water being available for hydration, as well as a more porous cementitious matrix. The densest accumulations of aggregates in the concrete were destroyed, and the interfacial transition zone of the RAC was deteriorated, resulting in decreased concrete strength. In summary, in terms of the compressive strength of the MSRAC, the optimal rate of replacement of high–quality MS was 100%, the optimal rate of replacement of medium quality MS was 50–75%, and the optimal rate of replacement of low–quality MS was 25–50%.

### 3.3. Chloride Diffusion Coefficient of High––Strength MSRAC

**Figure 8:**
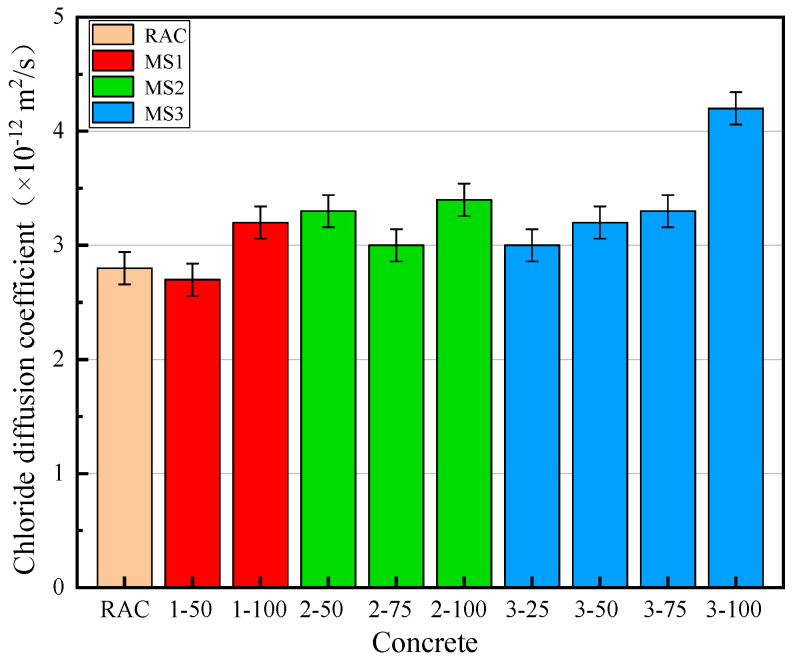
Chloride diffusion coefficient of concrete.

Figure 8 shows the chloride diffusion coefficient of MSRAC. It can be seen that the worse the MS quality and the higher the rate of replacement, the higher the chloride diffusion coefficient. When the replacement of MS was 100%, the maximum chloride diffusion coefficient occurred in the concrete containing MS3, and the minimum chloride diffusion coefficient occurred in the concrete containing MS1. For the MSRAC with MS1, the chloride diffusion coefficient of MS1–50 was slightly lower than that of RAC, while it increased by 14.3% when the rate of replacement with MS1 was 100%. For the MSRAC with MS2, the chloride diffusion coefficient of the concrete was greater than that of RAC. The chloride diffusion coefficients of MSRAC containing 50%, 75% and 100% MS2 were 17.9%, 7.1% and 21.4% higher than that of RAC, respectively. For the MSRAC with MS3, the chloride diffusion coefficient increased with increasing rate of MS3 replacement. The chloride diffusion coefficient of MSRAC containing 100% MS3 was 50% higher than that of RAC.

In RAC, the mortar and interfacial transition zone were weak areas that favored chloride penetration. On the one hand, due to the small particle size of stone powder, the stone powder was able to fill in the pores of the mortar and the interfacial transition zone, resulting in a closer accumulation of concrete particles. The number of open pores decreased and the chloride diffusion coefficient of the concrete decreased [40]. On the other hand, the mud powder content, represented by MBV, also affected the chloride penetration resistance of the concrete. When the mud powder content increased, the mud powder absorbed water, which was subsequently no longer available for cement paste hydration and workability, resulting in greater porosity, promoting chloride penetration [36]. When the positive effect is greater than the negative effect, the chloride diffusion coefficient increases. High–strength concrete contains a large amount of cementitious materials. Therefore, the density of concrete is high, and the accumulation of particles is close. The positive effect of stone powder as microfiller was not strong. The excess stone powder exerted a negative effect similar to that of mud powder. With decreasing MS quality, the stone powder content and MBV increased, resulting in an increase in the chloride diffusion coefficient.

The chloride diffusion coefficient limitation of concrete according to GB/T 50476–2019 [41] is shown in Table 7. The chloride diffusion coefficients of RAC prepared with medium– and high–quality MS at different rates of replacement all met the design requirements of the specification. Therefore, the maximum rate of replacement of medium– and high–quality MS based on their chloride penetration resistance was 100%.

**Figure 9:**
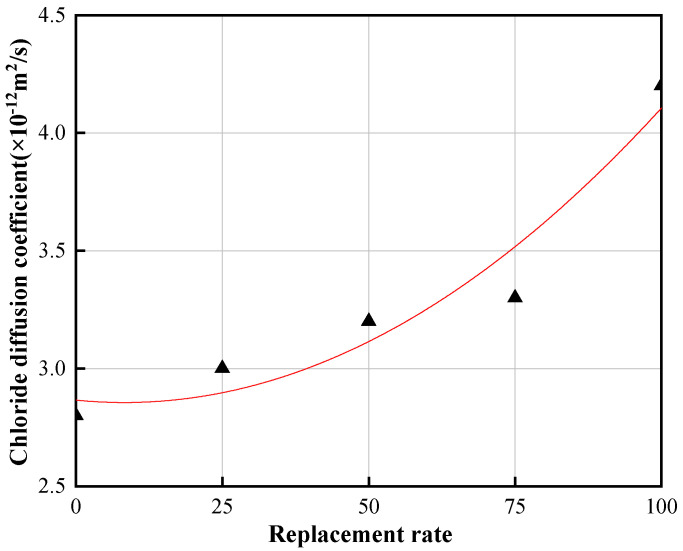
Fitted graph of chloride diffusion coefficient of MS3 series concrete.

The chloride diffusion coefficient of MS3–100 was $4{.2\;\times \;10}^{-12}{\;m}^{2}/s$. Therefore, through data fitting of MS3 series’ chloride diffusion coefficient, Equation (1) was obtained. It can be seen that the rate of replacement of low–quality MS had a quadratic parabolic relationship with the chloride diffusion coefficient, as shown in Figure 9. Substituting $D_{\mathrm{RCM}}{=4\;\times \;10}^{-12}m^{2}/s$ into Equation (1), the maximum rate of replacement of low–quality MS based on chloride penetration resistance was found to be 96.0%.

(1) $$y=1{.48571\;\times \;10}^{-4}x^{2}\;-\;0.00246x+2.86571,\;x\;\in \;\left[0,100\right]{,\;R}^{2}=0.933$$

where y is the chloride diffusion coefficient, and x is the rate of replacement.

### 3.4. Free and Bound Chloride Ion Content of High–Strength MSRAC

**Figure 10:**
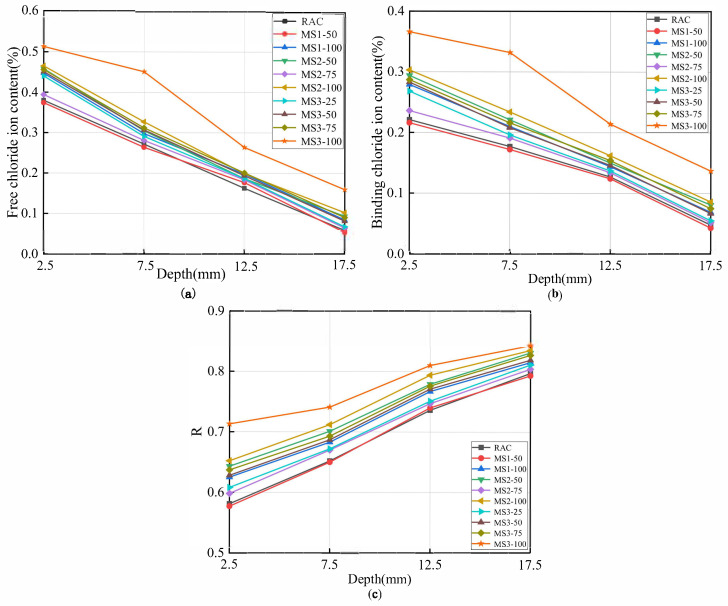
Relationship between chloride content and diffusion depth of concrete: (**a**) free chloride ion content; (**b**) bound chloride ion content; (**c**) chloride binding rate.

Figure 10a shows the relationship between the free chloride ion content of concrete and sample depth. It can be seen that the free chloride ion contents of RAC and MS1–50 at different depths were similar and were the lowest, which is due to the dense structures of RAC and MS1–50, restricting the invasion of chloride ions. The free chloride ion content of MS3–100 at each depth was the highest. This is because the concentrations of mud powder and stone powder in MS3 were significantly greater than those in RS, MS1 and MS2. These fine particles absorbed free water, reduced workability and cement hydration, increased porosity, and provided channels for chloride ion penetration. Therefore, the free chloride ion content of MS3–100 was the highest. Free chloride ion is the main cause of steel corrosion and bearing capacity decline in concrete. The passive film on the surface of steel bars breaks, and pitting corrosion occurs as a result of chloride ion corrosion, resulting in the generation of ferric hydroxide, the volume of which expands by 3–4 times, resulting in expansion and cracking of concrete protective layer [42].

Chloride ion binding in concrete is the phenomenon whereby chloride ion binds with cement hydration products, including through physical adsorption and chemical bonding. The physical adsorption of chloride ions is due to electrostatic or van der Waals forces attached to the pore wall or hydration products. This combination is unstable, and chloride ions can easily become free [43]. Chemical bonding is the interaction between chloride ions and hydration products in the form of chemical bonds. On the one hand, chloride ions react with calcium aluminate hydrates (C–A–H) to form Friedel’s salt (3CaO•Al_2_O_3_•CaCl_2_•10H_2_O). On the other hand, they react with calcium hydroxide in the pores to form expansive compound salt (CaCl_2_•Ca(OH)_2_•2H_2_O) [44]. Figure 10b,c show the trends in the variations of bound chloride ion content and chloride ion binding rate with sample depth. It can be seen that the bound chloride ion content and chloride ion binding rate at the same depth increased with decreasing MS quality and increasing rate of replacement. This is mainly due to the high stone powder and mud powder content. These powders absorbed free water, which was then no longer available for cement paste hydration and workability, subsequently resulting in greater porosity, destroying the densest accumulation of aggregates in the concrete and resulting in an increase in the porosity and specific surface area of mortar. The binding ability of the concrete to chloride ions was improved, leading to high bound chloride ion content and chloride ion binding rate. Additionally, it can be seen from Figure 10b that there was a decrease in the bound chloride ion content with increasing sample depth. In Figure 10c, the chloride ion binding rate increased with increasing sample depth. The reason for this is that the penetration depth of chloride ion into concrete is limited. With increasing sample depth, the free chloride ion content decreased, resulting in less bound chloride ions. Finally, the free chloride ion content gradually became equal to the bound chloride ion content. Therefore, the bound chloride ion content decreased, and the chloride binding rate increased. Previous studies have shown that the bound chloride ion content in concrete is unstable. Due to the action of environmental chloride ion concentration, acidification, chemical corrosion, external electric field, and temperature, the stability of bound chloride ion content can change, releasing free chloride ions [45,46]. Therefore, the influence of bound chloride ion content should be considered in the evaluation of the chloride penetration resistance of MSRAC.

Overall, the chloride ion content and chloride ion binding rate of the MSRAC were higher than those of the RAC. The lower the quality of MS and the higher the rate of replacement, the higher the chloride ion content and binding rate were. In addition, the chloride ion content of concrete was positively correlated with chloride diffusion coefficient.

Izquierdo et al. [47] showed that the critical free chloride ion content (in the mass fraction of cementitious material) of reinforcement ranged from (0.497 ± 0.26)% to (0.569 ± 0.177)%, and the critical total chloride ion content (in the mass fraction of cementitious material) ranged from (0.632 ± 0.112)% to (0.771 ± 0.346)%. It can be concluded from Figure 10a,b that at different sample depths, only the free chloride ion content of MS3–100 at 2.5 mm reached the critical value. The total chloride ion content of MS1–100, MS2–50, MS2–100 and MS3 series at 2.5 mm reached the critical value.

### 3.5. SEM Analysis

**Figure 11:**
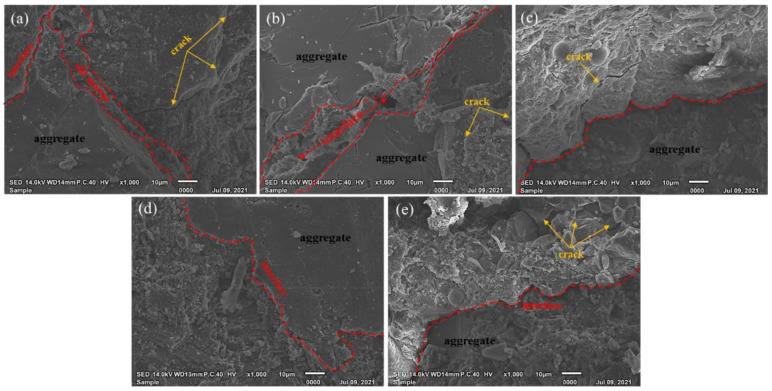
SEM images of RAC and MSRAC with medium– and low–quality MS before the RCM test: (**a**) RAC; (**b**) MS2–50; (**c**) MS2–100; (**d**) MS3–25; (**e**) MS3–100.

Figure 11 presents the SEM images of RAC and MSRAC with medium– and low–quality MS before the RCM test. Compared to RAC (Figure 11a), it can be seen that the microstructure of the MSRAC prepared with MS was looser, with MS3–100 being the looses. From Figure 11b–e, it can be seen that the microstructure of MSRAC became increasingly loose, accompanied by the formation of micro–cracks and pores, with increasing rate of replacement of medium– and low–quality MS. The bonding between mortar and aggregate was fragile, and the interfacial transition zone was fuzzy. Additionally, there were some micro–cracks around the interfacial transition zone. In this experiment, the quality and rate of replacement of MS were the determinants of MSRAC performance. The gradual deterioration of the microstructure indicated that the compressive strength and chloride resistance penetration of the MSRAC deteriorated with decreasing MS quality and increasing rate of replacement. This verified the macro law at the micro level.

**Figure 12:**
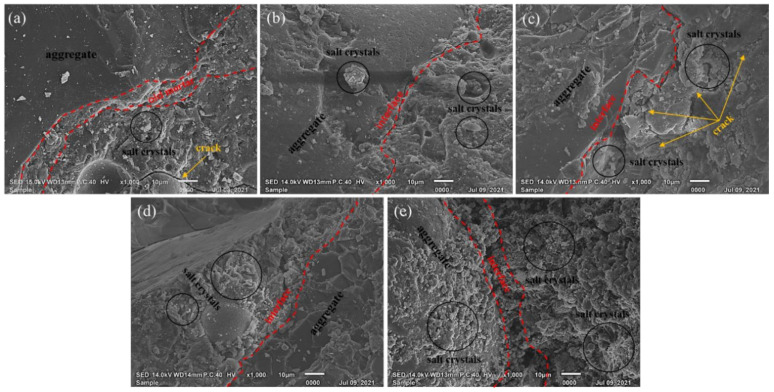
SEM images of RAC and MSRAC with medium– and low–quality MS after the RCM test: (**a**) RAC; (**b**) MS2–50; (**c**) MS2–100; (**d**) MS3–25; (**e**) MS3–100.

Figure 12 shows the SEM images of the RAC and MSRAC with medium– and low–quality MS after the RCM test. Compared to Figure 11, it can be seen that after the RCM test, the microstructure of the concrete had deteriorated further, resulting in more microcracks and pores, and the surface became rougher. Large amounts of stone and mud powder can be observed on the surface of MS2–100, MS3–25 and MS3–100. These fine particles absorbed free water for hydration, leading to blurring and refining of the interfacial transition zone, resulting in more microcracks in the interfacial transition zone. These microcracks decreased the combination of aggregate and mortar and provided more channels for chloride ion penetration. In addition, there were many white salt crystals on the concrete surface after the RCM test, especially on the surface of MS3–100. This indicated that after entering the concrete, the chloride ions underwent a chemical combination reaction with the hydration products, forming expansive compound salt. These two reactions consumed the hydroxyl ion inside the concrete. The pH value of the pore solution decreased, resulting in the decomposition of C–S–H. This deteriorated the pore structure of the concrete and decreased the compactness and stability of the concrete [48,49]. This also explained the reason for which MS3–100 possessed the highest chloride diffusion coefficient and chloride ion content.

## 4. Conclusions

Three quality levels of MS were used to prepare MSRAC with different rates of replacement. The effects of the three kinds of MS with different rates of replacement on the compressive strength and chloride ion permeability of high–strength RAC were investigated. The following conclusions can be drawn from the results of this study:

(1) The worse the quality of the MS, the lower the compressive strength of the MSRAC. The compressive strength of MSRAC with high–quality MS increased with increasing rate of replacement. However, for medium– and low–quality MS, the compressive strength of MSRAC first increased and then decreased with increasing rate of replacement.
(2) The chloride diffusion coefficient, and the free and bound chloride ion content of MSRAC increased with decreasing MS quality and increasing MS rate of replacement. Except for MS3–100, the chloride diffusion coefficient was lower than the standard limits of a working life of 50 years and 100 years when the chloride environmental action grade is D and E. The chloride diffusion coefficient of MSRAC with MS3 showed a quadratic parabola relationship with the rate of replacement. The maximum rate of replacement of MS3 in 100 years under environmental action level E was 96.0%.
(3) In light of the compressive strength and chloride penetration resistance, high–quality MS was able to completely replace RS, while the rates of replacement for medium– and low–quality MS were 50–75% and 25–50% in high–strength MSRAC, respectively.
(4) SEM analysis showed that an appropriate amount of stone powder can fill the micro cracks and interfacial transition zone of RAC. This can improve the compressive strength of the RAC. Conversely, excessive stone powder and mud powder absorbed free water when the concrete mixing is insufficient, resulting in the blurring of the interfacial transition zone and an increase in the number of microcracks and pores, having an unfavorable effect on compressive strength and chloride penetration resistance.

## Figures and Tables

**Table 1:** Quality of MS used in some references.

Reference	Stone Powder Content/%	MBV	Crushing Value/%	Soundness/%
Shen et al. [10]	III	I	III	–
	III	I	I	–
	III	III	I	–
	I	I	II	–
Shen et al. [11]	III	I	III	–
	III	I	I	–
	III	III	I	–
	I	I	I	–
	III	III	II	–
	II	III	III	–
	I	I	I	–
	I	I	II	–
Li et al. [14]	I	I	I	I
	II	I	I	I
	III	I	I	I
Shen et al. [18]	I	I	I	–
	I	I	I	–
	II	I	II	–
Tian et al. [20]	I	–	–	–
	III	–	–	–

Note: I, II and III represent high, medium and low quality, respectively.

**Table 2:** The composition of fly ash and silica.

Composition (%)	CaO	SiO_2_	Al_2_O_3_	FeO_3_	MgO	MnO	K_2_O	TiO_2_	SO_3_
Fly ash	3.72	51.50	29.33	3.77	1.16	0.22	1.70	0.98	1.69
Silica	0.27	87.03	1.12	0.97	0.88	0.14	–	–	0.86

**Table 3:** The properties of cement.

Cement Sort		P.O. 52.5
Blaine fineness (m^2^/kg)		382
Normal consistency (%)		27.8
Soundness		Qualified
Initial setting time (min)		187
Final setting time (min)		258
Flexural strength (MPa)	3 d	6.2
	28 d	9.3
Compressive strength (MPa)	3 d	30.8
	28 d	55.7

**Table 4:** Physical properties of MS.

MS	Standard Values—I	MS1	Standard Values—II	MS2	Standard Values—III	MS3
Fineness modules	–	2.8	–	3.2	–	2.3
Apparent density (kg/m^3^)	–	2737	–	2746	–	2654
Stone powder content (%)	0–10	7.0	0–10	10.2	10–15	15.6
MBV(g/kg)	0–0.5	0.5	0.5–1.0	0.9	1.0–1.4	1.7
Crushing value (%)	0–20	15.2	20–25	20.8	25–30	29.0
Soundness (%)	0–8	6.4	0–8	7.8	8–10	10.3
Water absorption(%)	–	1.2	–	1.8	–	2.0

Notes: Standard values—I, II and III represent high, medium and low quality, respectively.

**Table 5:** Physical properties of RCA.

Coarse Aggregate	Close Packing Density (kg/m^3^)	Apparent Density (kg/m^3^)	Crushing Value (%)	Water Absorption (%)
RCA	1477	2620	16%	6.2%

**Table 6:** Concrete mix design (kg/m^3^).

Sample	RS	MS	RCA	Cement	Fly Ash	Silica Fume	Water	Additional Water	Superplasticizer	Fresh Concrete Fluidity
RAC	609	0	1095	438	88	58	146	68	5.8	Good
MS1–50	304	318	1095	438	88	58	146	72	5.8	Good
MS1–100	0	636	1095	438	88	58	146	76	5.8	Slightly viscous
MS2–50	304	319	1095	438	88	58	146	73	5.8	Slightly viscous
MS2–75	152	478	1095	438	88	58	146	75	5.8	Slightly viscous
MS2–100	0	638	1095	438	88	58	146	78	5.8	Extremely viscous
MS3–25	456	154	1095	438	88	58	146	72	5.8	Slightly good
MS3–50	304	308	1095	438	88	58	146	74	5.8	Slightly viscous
MS3–75	152	462	1095	438	88	58	146	77	5.8	Extremely viscous
MS3–100	0	616	1095	438	88	58	146	80	5.8	Extremely viscous

**Table 7:** The chloride diffusion coefficient limitation of concrete [41].

Environment Action Classification	Design Working Life	
	50 Years	100 Years
D ^a^	${<10\;\times \;10}^{–12}{\;m}^{2}/s$	${<7\;\times \;10}^{–12}{\;m}^{2}/s$
E ^b^	${<6\;\times \;10}^{–12}{\;m}^{2}/s$	${<4\;\times \;10}^{–12}{\;m}^{2}/s$

^a^ Atmospheric region (mild salt fog): Atmosphere at sea more than 15 m above average water level; Outdoor environment on land within 100–300 m of the tidal shoreline; Mild sputtering environment of deicing salt; Contact with high concentration of chloride ion water and a dry–wet alternate environment. ^b^ Atmospheric region (heavy salt fog): Atmosphere at sea less than 15 m above average water level; Outdoor environment on land within 100 m of the tidal line and 15 m below the sea level; Tide and splash zones; Direct contact with de–icing salt solution environment; Heavy sputtering by deicing salt solution or heavy salt fog environment; Contact with high concentration of chloride solution and dry–wet alternate environment.

## Data Availability

The data presented in this study are available on request from the corresponding author.

## Abbreviations

MS	manufactured sand
NAC	natural aggregate concrete
RCA	recycled coarse aggregate
SEM	Scanning Electron Microscope
RCM	rapid chloride ions migration coefficient
C_3_S	Ca_3_SiO_5_
RAC	recycled aggregate concrete
MSRAC	manufactured sand recycled aggregate concrete
MMBV	multiplication of stone powder content and MBV
MBV	methylene blue value
RS	river sand
C–S–H	calcium silicate hydrates
